# Novel Techniques for *Ex Vivo* Expansion of Cord Blood: Clinical Trials

**DOI:** 10.3389/fmed.2015.00089

**Published:** 2015-12-11

**Authors:** Rohtesh S. Mehta, Katayoun Rezvani, Amanda Olson, Betul Oran, Chitra Hosing, Nina Shah, Simrit Parmar, Sue Armitage, Elizabeth J. Shpall

**Affiliations:** ^1^Division of Hematology, Oncology and Transplantation, University of Minnesota Medical Center, Minneapolis, MN, USA; ^2^Division of Cancer Medicine, Department of Stem Cell Transplantation, The University of Texas MD Anderson Cancer Center, Houston, TX, USA

**Keywords:** cord blood, *ex vivo* expansion, culture, cytokines, mesenchymal progenitor cells, SR1, nicotinamide, copper chelator, TEPA, Notch ligand, transplantation, CBT

## Abstract

Cord blood (CB) provides an excellent alternative source of hematopoietic progenitor cells (HPC) for patients lacking human leukocyte antigen-matched peripheral blood or bone marrow graft for transplantation. However, due to the limited cell dose in CB graft, it is associated with prolonged time to engraftment, risk of graft rejection, infections, and treatment-related mortality. To increase the cell dose, a variety of *ex vivo* expansion techniques have been developed. Results of traditional methods of CB expansion using cytokines alone were disappointing. Expanding CB cells with mesenchymal progenitor cells led to sizeable increase in graft content and improved engraftment. Other methods used HPC-differentiation blockers, such as nicotinamide analogs, copper chelators, inducing constitutive *Notch* signaling, or an aryl hydrocarbon receptor antagonist (StemReginin1). Many of these methods lead to substantial expansions of total nucleated cells and CD34^+^ cells, and significantly improved time to neutrophil or platelet engraftment in patients transplanted with the expanded products compared to the recipients of unmanipulated CBT. These studies differ not only in the expansion method but also with regards to the cytokines used, patient population, conditioning regimens, and transplantation practices, to name a few. Some of these methods employed expansion of a portion of CB unit in the setting of single CBT, while others in the setting of double CBT. Here, we review various procedures used for CB expansion and highlight some of the key differences. Novel methods of improving engraftment that aim at improving bone marrow homing potential of CB cells are not reviewed.

## Introduction

The numbers of allogeneic hematopoietic stem cell transplantation (HSCT) recipients in the United States currently exceeds 8000 a year and are consistently increasing (1). A suitable human leukocyte antigen (HLA)-matched donor can be identified for about 75% of Caucasian patients, but the probability is <20% for African-Americans and those belonging to other racial and ethnic minority groups (2). Umbilical cord blood (CB) provides an alternative source of stem cells for patients without HLA-matched peripheral blood (PB) or bone marrow (BM) donor. Transplantation with CB has several unique advantages, such as the ease of collection, minimal risk of infection transmission to the recipient, and no risk of donor attrition (3–5). Further, ready availability of CB units can reduce the time to transplantation by 4–5 weeks compared with those receiving HSCT from a matched unrelated donor (6). Rapid procurement of CB grafts is especially advantageous for patients who are in urgent need of transplantation. Moreover, the risks of disease relapse (7) and graft-versus-host disease (8–10) are lower after CB transplantation (CBT) compared with other donor sources. On the other hand, there is a higher risk of graft rejection, slower engraftment, and delayed immune reconstitution after CBT (7, 8, 11, 12).

## Why Do We Need to Expand CB Graft?

Many of the adverse effects after CBT are related to the low numbers of total nucleated cells (TNCs) and CD34^+^ cells present in a CB graft, which are typically only about 5–10% of the doses available for PB or BM transplants. Low cell doses negatively affect engraftment and other outcomes after CBT (13–22). Moreover, as transplantation using HLA-mismatched CB units is commonly performed, obtaining higher the numbers of TNC in a graft becomes even more crucial. In recipients of 1–2 HLA mismatched CBT, the best outcomes are noted after transplantation with grafts containing a median TNC dose of 10 × 10^7^/kg or higher, while the grafts containing <2.5 × 10^7^/kg TNCs or CD34^+^ dose of <1.7 × 10^5^ cells/kg lead to worse outcomes (17, 22). Although the use of double-unit CBT (DCBT) (23) is a relatively straightforward technique and almost doubles the available graft dose instantaneously, it does not increase the speed of engraftment compared with the recipients of adequately dosed single CBT, or that observed with other graft sources (23, 24).

## *Ex Vivo* Expansion of Cord Blood Cells

A distinct approach to increase cell dose is to expand CB hematopoietic progenitor cells (HPCs) *ex vivo* prior to infusion. Expansion of CB units can augment the numbers of colony-forming unit–granulocyte-macrophages (CFU-GMs), which are higher in CB compared with PB or BM (25, 26). Moreover, the proliferative ability of CB HPCs is higher than their BM counterparts (27–30). Therefore, clinical grade expansion of CB cells with a variety of techniques were attempted, such as (1) using cytokines alone in culture, (2) blocking *in vitro* differentiation of early progenitor cells (EPCs) using a variety of methods like (a) copper chelator, (b) nicotinamide (NAM) analog, (c) aryl hydrocarbon receptor antagonist, and (d) inducing constitutive *Notch* signaling, or (3) co-culturing CB cells with mesenchymal progenitor cells (MPCs). Some of these studies expanded a fraction of a CB unit in the setting of single-unit CBT, while others in the setting of DCBT. Some authors started with an unselected CB cell population for expansion, while others used a selected subset, such as CD34^+^ or CD133^+^ cells. Moreover, conditioning regimens, use of antithymocyte globulin, patient population, and disease characteristics vary among studies.

## Static Culture with Cytokines

The application of cytokine-derived “static culture” expansion was tested in a study of 37 patients (median age 38 years, median weight 61 kg) with various malignancies. In this study, CD34^+^ cells were purified from 40 to 60% fraction of a CB graft, and then expanded in cultures supplemented with stem cell factor (SCF), granulocyte colony-stimulating factor (G-CSF), and megakaryocyte growth and differentiation factor for 10 days (31). In the first cohort, the unexpanded fraction of CB unit was infused immediately on the day of thaw while the expanded fraction was infused on day 10 after expansion. In the second cohort, both the expanded and the unexpanded fractions were infused 10 days following the initial thaw. The results were rather discouraging primarily the Isolex 300-i device (Nexell, Irvine, CA, USA) and anti-CD34 antibody used for CD34^+^ selection resulted in significant upfront loss of cells. This resulted in very low numbers of CD34^+^ cells available for expansion and subsequent infusion (median CD34^+^ cell dose 0.89 × 10^5^/kg and median TNC count, 0.79 × 10^7^/kg). The median time to neutrophil was 28 days (range, 15–49 days) and of platelet engraftment of greater than 20 × 10^9^/L was 106 days (range, 38–345 days) (31).

## Continuous Perfusion Method with Cytokines

Another technique utilizing cytokines for expanding CB cells was reported by the Duke University Medical Center using an advanced computer automated “continuous perfusion” culture method (32). The study used the Aastrom Replicell bioreactor, which constantly monitored culture conditions and perfused growth medium with cytokines including PIXY321 [granulocyte-macrophage colony stimulating factor (GM-CSF)/interleukin (IL)-3 fusion protein], erythropoietin (EPO), and flt-3 ligand for 12 days. The CB units were thawed on day 0; the unmanipulated fraction was infused on the same day, while a small fraction of the graft was infused 12 days after expansion. The study included pediatric patients with a median age of 4.5 years and median weight of 17 kg. This method resulted in expansion of TNCs by a median of 2.4-fold, leading to identical median infused TNC doses of the unmanipulated and the expanded fractions (2.05 × 10^7^/kg). Nonetheless, the expansion of CD34^+^ cells was modest (median of 0.5-fold), resulting in low infused doses of CD34^+^ cells (unmanipulated fraction, 0.78 × 10^5^ cells/kg and expanded fraction, 0.10 × 10^5^ cells/kg). The median time to neutrophil engraftment was 22 days (range, 13–40 days) and that of platelet engraftment of greater than 50 × 10^9^/L was 94 days (range, 41–370 days), which were not different from what is expected in the recipients of unmanipulated grafts. Moreover, 3 out of 27 patients had graft failure.

## Blocking Differentiation of Cord Blood Early Progenitor Cells

It is noteworthy from the previous study that despite an actual increase in TNCs with CB expansion, the rapidity of engraftment was not improved. This is conceivably secondary to the expansion of “lower-quality” short-term reconstituting HPCs and cytokine-induced differentiation of EPCs (CD34^+^ CD38^−^ or CD34^+^ Lin^−^) into cells of committed lineage (CD34^+^ Lin^+^) that have poor BM homing capabilities (33–37). Therefore, several methods were designed to expand EPCs while blocking their differentiation, with a goal of selectively expanding cells that possess better BM homing capabilities.

### Copper Chelator

A polyamine copper chelator tetraethylenepentamine (TEPA) can block cytokine-induced differentiation of EPCs and expand them without affecting subsequent differentiation and proliferation of mature committed cells (36, 37). A preclinical study tested this technique by selecting CB CD133^+^ cells and culturing them with TEPA and “early acting cytokines,” namely flt-3 ligand, IL-6, thrombopoietin (TPO), and SCF for 3 weeks. This resulted in an impressive expansion of CD34^+^ cells by 89-fold, CD34^+^ CD38^−^ by 30-fold and colony forming units (CFU) by 172-fold. The expanded cells demonstrated improved marrow engraftment potential in sub-lethally irradiated non-obese diabetic (NOD/SCID) mice compared with that of unexpanded cells. Moreover, the expanded cells maintained the potential to differentiate into various hematopoietic lineages *in vivo* (37). Based on these encouraging results, a phase I–II study tested this method in 10 patients with hematological malignancies who underwent single unit CBT (median age 21 years, median weight 68.5 kg). One patient failed to engraft. In nine evaluable patients, the median time to neutrophil engraftment was 30 days (range, 16–46 days) and that of platelets was 48 days, which again were no better than the results expected in recipients of unmanipulated CBT. A subsequent large prospective trial reported results of 101 patients (median age 37 years, median weight 68 kg) who underwent single CBT in comparison with DCBT controls (*n* = 295) from the Center for International Blood & Marrow Transplant Research (CIBMTR) and the Eurocord registries. Owing to 400-fold TNC and 77-fold CD34^+^ expansion, the median infused doses of TNC was 2.2 × 10^7^/kg and that of CD34^+^ cells was 9.7 × 10^5^/kg. The median time to neutrophil (21 vs. 28 days, *P* < 0.0001) and platelet engraftment (54 vs. 105 days, *P* = 0.008) as well as day 100 overall survival (84.2 vs. 74.6%, *P* = 0.035) were significantly improved compared with the controls (38).

### *Notch* Signaling

Another method of blocking EPC differentiation is the induction of constitutive *Notch* signaling, which can establish immortalized cell lines that can be cultured in liquid medium with SCF, IL-6, IL-11, and flt-3 ligand, with continued undifferentiated proliferation for over 8 months. More importantly, these cells retain the capability to differentiate into either myeloid cells when stimulated with GM-CSF or lymphoid cells in the presence of SCF, IL-3, and IL-7 (39). Delaney et al. (40) reported the preliminary results of their study using this technique, where CD34^+^ selected CB cells were transduced with an engineered *Notch* ligand (Delta1^ext-IgG^) and cultured for 16 days in the presence of IL-3, IL-6, TPO, SCF, and flt-3 ligand, leading to an impressive 222-fold average expansion of CD34^+^ cells. The expanded cells had enhanced repopulating ability *in vivo* when infused into NOD/SCID mice compared with the control mice. Preliminary results of an ongoing phase I study were reported in 10 patients (median age 27.5 years, median weight 61.5 kg) who underwent DCBT following total body irradiation (TBI)-based myeloablative conditioning. The expanded unit contained a median TNC dose of 4.6 × 10^7^/kg and CD34^+^ cell dose of 60.3 × 10^5^/kg. The respective cell doses in the unmanipulated unit were 3.3 × 10^7^/kg and 2.4 × 10^5^/kg. The expanded CB unit was infused 4 h after the infusion of unmanipulated unit. No infusional toxicities were noted; one patient had primary graft failure. In nine evaluable patients, neutrophil engraftment occurred at a median of 16 days (range, 7–34 days), compared with 26 days (range, 16–48 days) in a concurrent cohort of 20 patients who received two unmanipulated CB units, *P* = 0.002. Furthermore, eight of the nine engrafted patients had evidence of sustained chimerism derived from the expanded CB unit. In one patient, partial chimerism (10–15%) from the expanded cord was noted for up to 240 days post-DCBT. At a median follow-up 354 days, 70% of the patients were in complete remission with sustained engraftment. Nevertheless, the expanded cells did not persist beyond 1 year, after which the unexpanded CB unit completely contributed to engraftment (40).

### Nicotinamide

Another technique of inhibiting the differentiation of EPCs involves the use of NAM. A sirutin family of protein deacetylases (SIRT1) plays an important role in determining lifespan of a number of lower organisms (41) and regulates apoptosis and self-renewal in mouse embryonic stem cells by deacetylating p53 (42). Inhibition of a SIRT1 deacetylase enhances proliferation potential of HPCs with decreased dependency on growth factors and increases telomerase activity via catalytic subunit of telomerase, hTERT (43). NAM is a potent and specific inhibitor of SIRT1; it is known to inhibit differentiation of HPCs and plays an important role in adhesion, migration, and proliferation of stem cells (44). In a pre-clinical study, culturing CB CD34^+^ cells with SCF, TPO, IL-6, and flt-3 ligand with or without NAM resulted in about 40-fold expansion of CD34^+^ cells in both the groups. However, the EPCs (CD34^+^Lin^−^ cells) expanded to a greater degree in NAM-treated cells. Further, transplantation of carboxyfluorescein diacetate succinimidyl ester (CFSE)-labeled cells into irradiated NOD/SCID mice showed that the NAM-treated cells had significantly improved homing capacity and engraftment potential compared with the untreated cells (45). A phase I trial included 11 adult patients (median age 45 years, median weight 83 kg) who underwent DCBT using TBI-based myeloablative conditioning. One CB unit was thawed on day 21 and selected for CD133^+^ cells using immunomagnetic beads. The negative fraction was cryopreserved, while the positive fraction was cultured with NAM, SCF, TPO, and IL-6 in culture bags for 21 days. The CD133-negative fraction was later thawed on day 0 and infused after the infusion of cultured fraction. The infusions of expanded and unmanipulated CB units were separated by 2 h. After 3 weeks of culture, TNCs expanded by a median of 486-fold and CD34^+^ cells expanded by a median of 72-fold. The final total infused TNC dose was 3.1 × 10^7^/kg and that of CD34^+^ was 35.0 × 10^5^/kg (including 0.7 × 10^5^/kg was from unmanipulated unit). Seven of the 10 patients attained partial or complete chimerism from the expanded unit, which persisted for up to 36 months in some cases. Two patients had long-term engraftment from the unmanipulated unit and one patient had graft failure. In evaluable patients, the median time to neutrophil engraftment was 13 days (range, 7–26 days) compared with 25 days (range, 13–38 days) in their historical controls (*P* < 0.001). The median time to platelet engraftment was 33 days (range, 26–49 days) compared with 37 days (range, 20–66 days) in historical controls (*P* = 0.085) (46).

### Aryl Hydrocarbon Receptor Antagonism

The University of Minnesota group presented the results of a phase I/II study of CB expansion using StemReginin1 (SR1), an aryl hydrocarbon receptor antagonist, which helps in the proliferation of CD34^+^ cells without differentiation in the presence of SCF, flt-3 ligand, TPO and IL-6 (47). A total of 17 patients with hematological malignancies underwent TBI-based myeloablative conditioning followed by DCBT, where 1 U was expanded *ex vivo* while the other was infused unmanipulated. The culture led to 328-fold median expansion of CD34^+^ cells, resulting in a median total infused CD34^+^ dose of 123 × 10^5^/kg. There were no graft failures. The median time to neutrophil engraftment was shorter in 11 patients in whom the SR1-expanded cord predominated (11 days) compared with patients in whom the unmanipulated cord predominated (23 days). The time to neutrophil and platelet engraftment were both significantly faster in the recipients of expanded CB as compared with 111 recipients of unmanipulated DCBT. More interestingly, two additional patients received only one SR1-expanded CB unit, with CD34^+^ doses of 250 × 10^5^/kg and 180 × 10^5^/kg; with neutrophil engraftment occurring in 12 and 8 days, respectively (47). The authors are currently testing the feasibility of transplantation with single expanded CB unit.

## Mesenchymal Progenitor Cell-Supported Expansion

All of the methods reviewed above require selection of either CD34^+^ or CD133^+^ cells. As seen with some of the techniques, the selection process itself can lead to sizeable degree of upfront cell loss ranging from 4 to 70%. One approach to circumvent this issue is to co-culture unselected CB cells with MPCs. As MPCs are components of *in vivo* hematopoietic microenvironment and produce cytokines and other proteins that regulate cell proliferation and homing (48), co-culturing CB cells with MPCs putatively creates an *ex vivo* stem cell “niche” (49, 50). Moreover, MPCs do not express HLA-class II histocompatibility antigens and thus can be obtained either by harvesting BM of a haploidentical family member or by clinical-grade “off-the-shelf” sources, such as Mesoblast (Mesoblast Limited, Melbourne, VIC, Australia) (51).

The efficacy of MPC-expanded CB cells was reported by the investigators from the MD Anderson Cancer Center in 31 adult patients (median age 31–39 years, median weight 75–79 kg) who underwent DCBT after myeloablative conditioning (52). The MPCs were obtained from a haploidentical family member for the first seven patients. However, due to the time required for generating MPCs from a family member and the logistics of it, “off-the-shelf” MPCs (Mesoblast) were used for subsequent 24 patients. The CB unit containing lower dose of TNC was thawed 2 weeks before transplantation, placed in cultures containing MPCs and supported with a cytokine cocktail of SCF, flt-3 ligand, TPO and G-CSF. After 14 days of culture, the non-adherant cells were removed, washed and infused after the infusion of freshly thawed unmanipulated CB unit. The culture resulted in 12.2-, 30.1-, and 17.5-fold increases in the TNC, CD34^+^ cells, and CFUs populations, respectively, which yielded final median doses of 5.84 × 10^7^/kg for TNCs, 9.7 × 10^6^/kg for CD34^+^ cells and 3 × 10^6^/kg for CFUs. No differences were observed in the expansion achieved from family member-derived or “off the shelf” MPCs. The median time to neutrophil engraftment was 15 days (range, 9–42 days) compared with 24 days (range, 12–52 days) observed in the CIBMTR controls, *P* < 0.001. The median time to platelet engraftment was 42 days (range, 15–62 days) compared with 49 days (range, 18–264 days) in the controls, *P* = 0.03. The cumulative incidence of neutrophil engraftment was also significantly improved in the study population (96%) compared with the controls (78%), *P* = 0.005. The cumulative incidence of platelet engraftment by day 180 was 75% in the study population compared with 46% in the controls, *P* = 0.01. There were no infusion related toxicities. All patients attained complete donor chimerism from one or the other cord between days 21 and 30. Interestingly, 54% of the patients attained hematopoiesis solely from the unmanipulated CB unit and 46% had mixed chimerism from both the units (52).

## Conclusion

The field of *ex vivo* expansion of CB graft has progressed remarkably. Beginning from the traditional culture methods using cytokines alone, the field witnessed noteworthy transformation to more elegant techniques, such as co-culturing of CB cells with MPCs and the use of EPC differentiation blockers like copper chelator (TEPA), NAM analogs, StemReginin1, and the *Notch*-ligands. Many of these techniques have improved the time to neutrophil engraftment appreciably, which is comparable to that seen with other donor types. However, all of these techniques require several days of culture before the final product is available for transplantation and these procedures can be performed only at specialized centers. A completely different approach to enhance engraftment focuses on increasing the homing capacity of CB cells to BM. These include the use of prostaglandin E2 analogs (53) and fucosylation (54). Such novel methods are quick – requiring only 30–120 min of incubation of CB cells prior to transplantation and have demonstrated significant improvements in engraftment in clinical trials. Future studies may test a combination of these approaches in order to shorten the culture time or to further enhance engraftment. Prospective comparative trials will also shed more light on the relative efficacy of these promising technologies.

## Author Contributions

RM and ES wrote the manuscript. All authors edited and approved the final version.

## Conflict of Interest Statement

The authors declare that the research was conducted in the absence of any commercial or financial relationships that could be construed as a potential conflict of interest.
